# Optimal Parameter Selection for Support Vector Machine Based on Artificial Bee Colony Algorithm: A Case Study of Grid-Connected PV System Power Prediction

**DOI:** 10.1155/2017/7273017

**Published:** 2017-08-22

**Authors:** Xiang-ming Gao, Shi-feng Yang, San-bo Pan

**Affiliations:** ^1^School of Physics and Electrical Engineering, Anyang Normal University, Anyang 455000, China; ^2^College of Electronic Information and Automation, Tianjin University of Science & Technology, Tianjin 300222, China

## Abstract

Predicting the output power of photovoltaic system with nonstationarity and randomness, an output power prediction model for grid-connected PV systems is proposed based on empirical mode decomposition (EMD) and support vector machine (SVM) optimized with an artificial bee colony (ABC) algorithm. First, according to the weather forecast data sets on the prediction date, the time series data of output power on a similar day with 15-minute intervals are built. Second, the time series data of the output power are decomposed into a series of components, including some intrinsic mode components IMFn and a trend component Res, at different scales using EMD. The corresponding SVM prediction model is established for each IMF component and trend component, and the SVM model parameters are optimized with the artificial bee colony algorithm. Finally, the prediction results of each model are reconstructed, and the predicted values of the output power of the grid-connected PV system can be obtained. The prediction model is tested with actual data, and the results show that the power prediction model based on the EMD and ABC-SVM has a faster calculation speed and higher prediction accuracy than do the single SVM prediction model and the EMD-SVM prediction model without optimization.

## 1. Introduction

With the increasing scale of grid-connected PV systems, the adverse effects of intermittent and uncertain characteristics of the PV system on the public grid are becoming increasingly important [1–3]. If the changes in the PV system power generation can be accurately predicted, a reasonable power grid scheduling and balanced power load configuration can be achieved to protect the security and stability of the public grid system. Currently, there are two methods to predict the output power of PV systems: the indirect and direct prediction methods. The crux of the indirect method is to predict the solar radiation intensity of the PV installation site, predict the solar radiation intensity at a certain time, and substitute it into the corresponding output model, thus obtaining the predicted output power value of the PV system [4]. Direct prediction methods do not require solar irradiance data and can predict the power output of PV power generation systems in the next time period by using only the historical PV system data and public weather information [5–8]. Some studies have shown that the influence of meteorological factors on the output power of PV systems is significant. If the meteorological conditions are similar in two time periods, the power output curves will have a great similarity. Therefore, it is possible to predict the output power of the grid-connected PV system by selecting a date with similar data [9].

The direct prediction method predicts the future power output by using the historical data of the output power based on a mathematical statistics prediction theory and method. The indirect prediction method is also called the step-by-step prediction method. In this method, the solar irradiance is forecasted, and the output power is then calculated based on the photoelectric conversion model. This method cannot obtain the output power directly, so it is called the indirect prediction method.

The above PV power prediction methods have their own characteristics and associated limitations in their application [10]. At present, the PV power prediction error resulting from a single prediction method is large, generally 15% to 30%, because the output power of a PV system is largely affected by meteorological factors. Furthermore, there are intermittent problems and uncertainties in photovoltaic power generation systems. The limitations of the prediction methods are also a key factor that causes a relatively large error. Numerous studies have shown that the accuracy of a single prediction method cannot meet the prediction accuracy requirements for the power generation of PV systems. Combined prediction methods can synthesize the advantages of multiple prediction approaches and improve the prediction accuracy of generated power of the PV system.

The empirical mode decomposition (EMD) has been largely and successfully combined to predict the nonlinear stochastic time series. This prediction method first decomposes the time series into multiple series of different frequencies, establishes prediction models for different series to reduce the interaction among the information about different characteristics, and finally reconstructs the prediction results to obtain the predicted value of the original series.

In this paper, a combined prediction model is introduced and applied to PV power prediction. The advantages of different algorithms are combined to establish and test the prediction model for the output power of grid-connected PV systems based on the EMD and ABC-SVM. This model effectively overcomes the defects, such as poor generalization performance, low prediction accuracy, and unstable prediction results, that are observed when a single model is adopted and successfully applies the artificial bee colony optimization algorithm and EMD method to predict the output power of grid-connected PV systems.

Support vector machine (SVM) is a novel machine learning method that is based on statistical learning theory and minimized structural risk. It has been successfully applied in nonlinear regression predictions in various fields, such as wind speed forecasting, short-term load forecasting, and tourist flow forecasting [11–13]. These results have proven that the SVM can successfully solve prediction problems with small samples, nonlinearity, and high dimensionality. However, the parameter optimization in SVM plays a crucial role in improving the prediction accuracy and stability. Therefore, it is vital to select the most appropriate parameter value for the SVM. Determining the optimal parameters for SVM is very important.

The artificial bee colony (ABC) technique is an optimization algorithm based on the intelligent foraging behavior of a honey bee swarm. The unique mechanism of division of labor and collaboration in the ABC algorithm makes bees collaborate in accordance with different search strategies to complete the task of seeking the optimization, showing strong global optimization seeking ability. The algorithm has been shown to be superior to the performance of the genetic algorithm, ant colony algorithm, and particle swarm algorithm in related research [14–18]. Therefore, the artificial bee colony technique is used to search for the optimal parameters of the SVM in this paper.

## 2. Clustering Selection Method of Similar Days for the Output Power of a PV System

**Figure 1:**
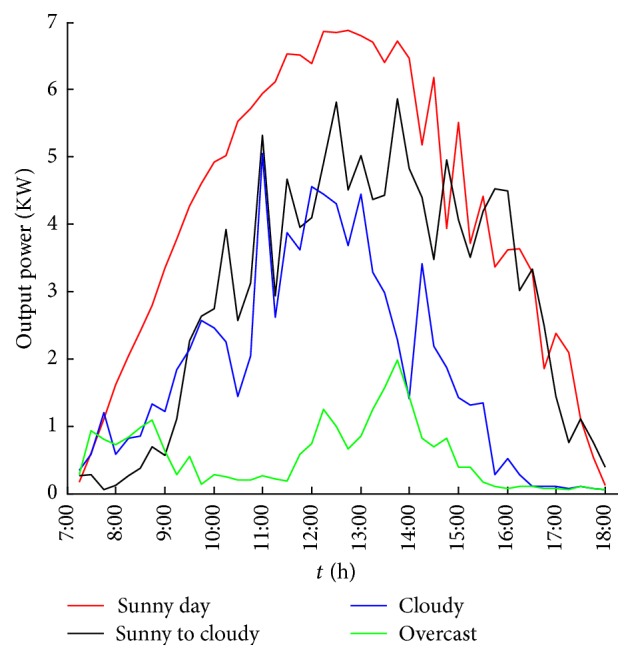
15-minute average output power output under different weather conditions.

**Figure 2:**
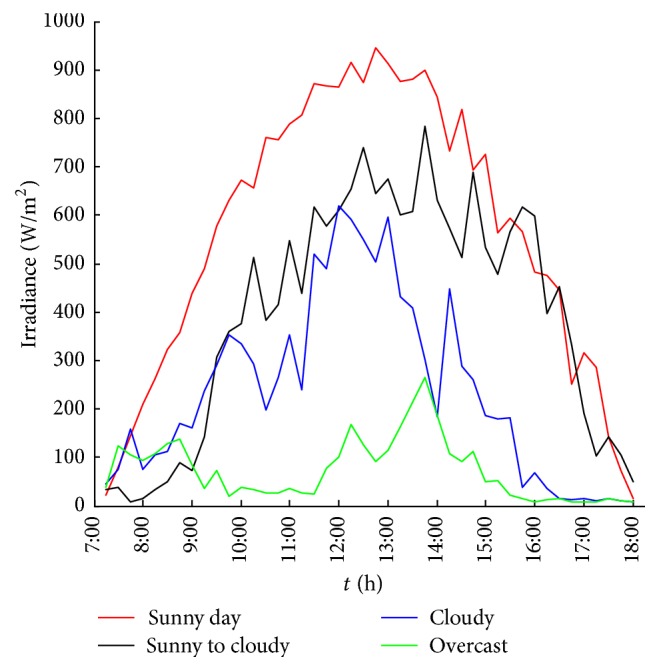
15-minute average irradiance under different weather conditions.

**Figure 3:**
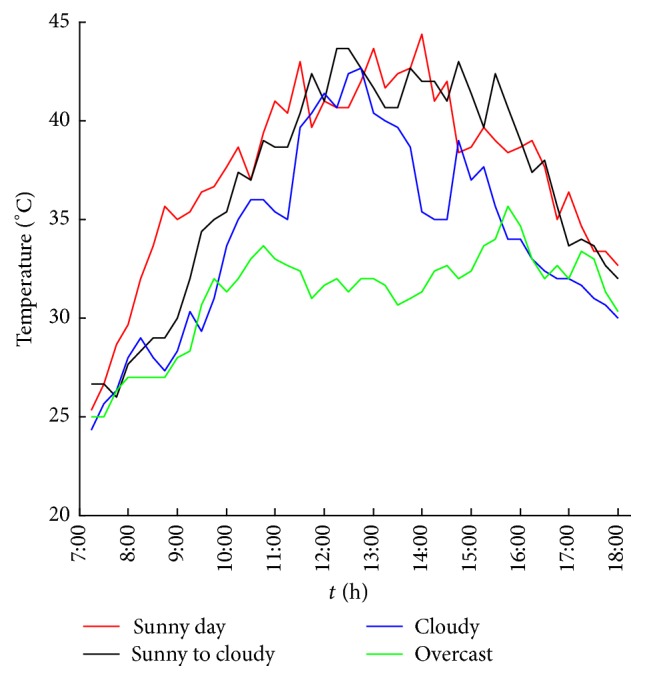
15-minute average temperature of the solar panels under different weather conditions.

It is found that the influence of meteorological factors on the output power of photovoltaic power generation system is significant. Under the same conditions, the size and the changing tendency of the output power will differ because of the varying weather types.  Figure 1 shows the change in the 15-minute output power of a 10 kW PV system under different weather types, sunny, sunny to cloudy (cloudy to sunny), cloudy, and overcast, in August 2014. Figures 2 and 3 show 15-minute average irradiance and temperature of the solar panels under different weather conditions similar to Figure 1.

The 10 kW PV array consists of 51 photovoltaic modules (PLUTO195-ade). These modules are connected both in series and in parallel to obtain a larger output power. Seventeen modules are connected in series, and 3 strings of series-connected modules are connected in parallel (17*∗*3*∗*975 W = 9.945 kW).

The manufacturer specifications for one module are as follows: open-circuit voltage (*V*oc) is 45.4 V, short-circuit current (*I*sc) is 5.52 A, and the voltage and current at maximum power (*V*mp and *I*mp) are 37.6 V and 5.19 A, respectively.

When the output power of the grid-connected PV system is predicted, finding reasonably similar days from the actual historical data can greatly improve the prediction accuracy of the output power of the PV system.

To find days that have similar weather and seasonal types and thereby determine temperature and humidity of the prediction day, we first classify the weather and seasonal types. The weather types are classified as sunny, sunny to cloudy (cloudy to sunny), cloudy, overcast, and rainy (snowy) which are represented by 1, 2, 3, 4, and 5, respectively. The seasonal types are classified as follows: March, April, and May for spring; June, July, and August for summer; September, October, and November for fall; and December, January, and February for winter. Then, the classification is combined with the historical data according to the seasonal types of the prediction day, and fuzzy clustering is carried out using the fuzzy *C*-mean algorithm. Finally, days similar to the prediction day are found. The general steps are as follows.

(1) Identify the clustering indexes. The weather type is *w*, the daily highest temperature is *T*^h^ and the lowest is *T*^l^, and the daily maximum humidity is *H*^h^ and the minimum is *H*^l^. To increase the comprehensiveness of the clustering samples, we take the historical meteorological data of the same season two years before as the clustering sample set. Assuming that the data set has *N* days, the sample set can be written as *D* = {*d*_*j*_∣*j* = 1,2,…, *N*}, where *d*_*j*_ = {*w*_*j*_, *T*_*j*_^h^, *T*_*j*_^l^, *H*_*j*_^h^, *H*_*j*_^l^}. At the same time, the number of clusters, *C*, the fuzzy weighting parameter, *m*, the threshold value, *ε*, and the initial iteration step, *L*, are determined.

(2) According to the determined *C*, vectors of *C* are randomly selected as the clustering center; that is, *v*^o^ = {*v*_*i*_∣*i* = 1,2,…, *C*}.

(3) Calculate the membership matrix *U*^*L*^ = |*μ*_*ij*_|_*C*×*n*_, where *μ*_*ij*_ represents the membership of vector *d*_*j*_ on class *v*_*i*_ and *μ*_*ij*_ satisfies the following:

(1) $$\begin{array}{c} \mu_{ij}=\left\{\begin{array}{cc} \overset{C}{\underset{k=1}{\sum }}{\left(\frac{r_{ij}}{r_{kj}}\right)}^{2/\left(m-1\right)}, & \text{if}\;r_{ij}\neq 1\\ 1, & \text{if}\;r_{ij}=1, \end{array}\right. \end{array}$$

where 1 ≤ *i* ≤ *C*,  1 ≤ *j* ≤ *n*,  *r*_*ij*_ represents the similarity between *d*_*j*_ and the clustering center *v*_*i*_, and *s* is the vector dimension.

(4) Calculate the clustering center *V*^*L*+1^ according to the following:

(2) $$\begin{array}{c} v_{i}=\frac{\sum_{j=1}^{n}{\left(\mu_{ij}\right)}^{m}x_{j}}{\sum_{j=1}^{n}{\left(\mu_{ij}\right)}^{m}},\;1\leq i\leq C. \end{array}$$

(5) Determine whether the termination condition is satisfied. If ‖*V*_*L*+1_ − *V*_*L*_‖ ≤ *ε*, the algorithm is terminated and the partition matrix, *U*, and the clustering center, *v*, are obtained. Otherwise, return to step (4) to continue the calculation until the termination condition is satisfied.

After the clustering is complete, the prediction day in the same class and its similar historical date can be obtained according to the partition matrix *U*.

## 3. Decomposing the Output Power Signal of the Grid-Connected PV System by EMD

The essence of EMD is to smooth the nonlinear and nonstationary signals based on local characteristic scale. The EMD also decomposes different scales of fluctuations or trends step-by-step from the original complex signals to form a series of intrinsic mode function (IMF) characteristics with different scales and a trend component [19–23]. Compared with the wavelet transform, the empirical mode decomposition (EMD) not only has the characteristic of multiresolution but also overcomes the difficulty in determining the scale of decomposition in the wavelet transform and selecting the wavelet base.

The empirical mode decomposition of *x*(*t*) and the time series of the PV output power are performed as follows.

(1) Identify all maximum points in the signal sequence composed of the PV output power in multiple similar days, and the upper envelope line *e*_*h*_(*t*) of the sequence is fit by interpolating the cubic spline function. Identify all minimum points in the output power time series, and the lower envelope line *e*_*l*_(*t*) of the sequence is fit by interpolating the cubic spline function.

(2) The average values of the upper and lower envelope lines are calculated as the average envelope line *e*_*v*_(*t*); a new data sequence *h*_1_(*t*) is obtained using the original power output time series *x*(*t*) minus the average envelope line *e*_*v*_(*t*). In general, *h*_1_(*t*) is a nonstationary time series that should be processed again based on the above discussion. Assuming that *h*_1*k*_(*t*) satisfies the IMF conditions after *K* treatments, the first IMF component imf_1_(*t*) is obtained, where imf_1_(*t*) = *h*_1*k*_(*t*), and it contains the shortest variable cycle component in the original output power time series.

(3) An output power time series *r*_1_(*t*) that removes the high-frequency component is acquired using the original output power time series *x*(*t*) minus the first IMF component imf_1_(*t*). We can obtain all of the IMF components and a trend component Res, as shown in the following formula, after continuing the above-mentioned smoothing treatment on *r*_1_(*t*).

(3) $$\begin{array}{c} r_{2}\left(\;t\;\right)=r_{1}\left(\;t\;\right)-{imf}_{2}\left(\;t\;\right)\\ r_{3}\left(\;t\;\right)=r_{2}\left(\;t\;\right)-{imf}_{3}\left(\;t\;\right)\\ \vdots \\ \mathrm{Res}\left(\;t\;\right)=r_{M-1}\left(\;t\;\right)-{imf}_{M}\left(\;t\;\right). \end{array}$$

Finally, the form of the decomposed PV output power time series *x*(*t*) is obtained:

(4) $$\begin{array}{c} x\left(\;t\;\right)=\overset{M}{\underset{i=1}{\sum }}{imf}_{i}\left(\;t\;\right)+\mathrm{Res}\left(\;t\;\right), \end{array}$$

where imf_*i*_(*t*) represents the intrinsic mode function component of the PV output power time series *x*(*t*) and Res⁡(*t*) represents the average trend component of the original signal sequence. Thus, the original PV output power time series can be decomposed into the sum of a series of intrinsic mode function components and an average trend component.

Taking the actual output power of the 10 kW grid-connected PV system of the new energy grid-connected PV power generation engineering technology center at a university of Henan Province as an example, the EMD decomposition of the output power time series with 15-minute intervals in 50 similar days is performed, and the results are shown in Figure 6. The output power time series is decomposed into seven IMF components and a trend component. IMF1 and IMF2 are the high-frequency components and have strong nonlinear and random change characteristics caused by abrupt changes in the weather. The frequencies of IMF3–IMF7 become significantly lower and show a strong periodicity, which is affected by meteorological factors; this is the main component of the output power. The residual component Res shows relatively gentle changes, has small amplitude, and is the minor component of the output power.

## 4. Artificial Bee Colony Algorithm and Support Vector Machine

### 4.1. Artificial Bee Colony Algorithm

The artificial bee colony (ABC) algorithm is a group intelligent optimization algorithm that simulates the process of bee colonies gathering honey [24]. The bees in the artificial bee colony algorithm can be divided into three types: employed bees, scout bees, and onlooker bees. The three types of bees cooperate with each other to complete different stages of the tasks in the honey mining process and identify the position of the optimal nectar source by collecting and sharing nectar sources. In the artificial bee colony algorithm, the optimal nectar source position corresponds to the optimal solution of a problem, and the amount of nectar contained in the nectar source corresponds to the fitness value of the solution.

After the initialization is complete, the employed bees search the neighborhood of the corresponding known nectar source (the original solution to a problem) and find a new nectar source (a new solution to the problem). The position of the new nectar source (the parameter value of the optimized problem) is determined according to the following:

(5) $$\begin{array}{c} v_{ij}=x_{ij}+\phi_{ij}\left(\;x_{ij}-x_{kj}\;\right), \end{array}$$

where *ϕ*_*ij*_ is a random number within [−1,1] that controls the generation range of the *x*_*ij*_ neighborhood, *k* ∈ {1,2,…, SN} and *j* ∈ {1,2,…, *D*} are randomly selected subscripts, and *k* is not equal to *i*.

SN employed bees return to the hive after completing the search task and share the searched nectar source information with the scout bees. The scout bees select the nectar source based on the amount of nectar in each source (fitness function value of a solution) and in accordance with the following:

(6) $$\begin{array}{c} p_{i}=\frac{{fit}_{i}}{\sum_{n=1}^{N}{fit}_{n}}. \end{array}$$

Subsequently, the scout bees will search near the selected nectar source and determine the position of a new nectar source per formula (5). They will use the method to select or not select a new nectar source, similar to nectar-gathering bees to determine whether to replace the old nectar source with the new one. If the nectar source *x*_*i*_ cannot be improved after it is updated for* limit* times, this nectar source will be discarded. The corresponding employed bees will also change to onlooker bees, which will reidentify a new nectar source in accordance with the following:

(7) $$\begin{array}{c} x_{i}=x_{min}+\mathrm{rand}\left(\;0,1\;\right)\left(\;x_{max}-x_{min}\;\right). \end{array}$$

The unique mechanism of dividing the labor and collaboration in the ABC algorithm makes bees work together with different search strategies to complete the task of seeking the optimized solution by showing a strong global optimization seeking ability.

### 4.2. Regression Model of SVM

Support vector machine can minimize the expected error and overcome the problem of overfitting because it is based on structural risk minimization principle. According to previous research, SVM can provide better resolutions for both classification and regression in different fields: fault classification, electricity load forecasting, wind speed forecasting, prediction of the air quality, and so on. The basic principle of SVM to solve regression prediction problems is described as follows. The sample set is normally denoted as

(8) $$\begin{array}{c} X=\left\{\;\left(\;x_{i},y_{i}\;\right)\;|\;x\in R^{n},\;y\in R,\;i=1,2,\ldots ,n\;\right\}. \end{array}$$

The regression model defines the functional relationship between *x*_*i*_ and *f*(*x*_*i*_) as

(9) $$\begin{array}{c} y=f\left(\;x_{i}\;\right)=w\cdot x_{i}+b, \end{array}$$

where *w*, *b* are the weight vector and threshold, respectively. Furthermore, the coefficients *w* and *b* can be found by solving the following convex quadratic programming problem:

(10) minw,b,ξi∗ 12w2+C∑i=1lξi∗+ξi

(11) S.t. yi−w·xi−b≤ε+ξi ξi∗,ξi≥0;i=1,2,…,n,

where *C* is penalty coefficient, *ξ*_*i*_^(*∗*)^ is slack variable and *ε* is the insensitivity coefficient. *ξ*_*i*_^(*∗*)^ guarantees the satisfaction of constraint condition; *C* controls the equilibrium between the complexity of model and training error; *ε* is a preset constant for controlling tube size. If *ε* is set too small, it will lead to overfitting; otherwise, it is easy to lead to the underfitting.

For nonlinear regression, assume that there is such a transform: Φ : *R*^*n*^ → *H*, *x* → Φ(*x*), making *K*(*x*, *x*′) = Φ(*x*) · Φ(*x*′), where *K*(*x*, *x*′) is called kernel function and (·) denotes inner product operation. When *K*(*x*, *x*′) satisfies the Mercer condition, it corresponds to the inner product of a transform space according to the functional theory. By introducing the Lagrange multipliers *a*_*i*_, *a*_*i*_^*∗*^, the nonlinear regression function can be determined (the detailed derivation procedure is shown in the supporting information in Supplementary Material available online at https://doi.org/10.1155/2017/7273017):

(12) y=fx=∑i=1lai∗−aiKxi,x+b

(13) S.t. ∑i=1lai∗−ai=0

In this study, we chose Gaussian radial basis function as the kernel function:

(14) $$\begin{array}{c} K\left(\;x,x^{\prime }\;\right)=\frac{\exp\left(-{\left.\left|x-x^{\prime }\right.\right|}^{2}\right)}{2\sigma^{2}}, \end{array}$$

where *σ*^2^ is the kernel parameter, and it precisely defines the structure of high dimensional feature space.

The penalty coefficient *C*, the insensitivity coefficient *ε*, and the kernel function parameter *σ*^2^ in SVM determine the accuracy and generalization performance of the algorithm.

## 5. Constructing a PV Power Prediction Model Based on EMD and ABC-SVM

The strong nonlinear and nonstationary power sequence signals by the grid-connected PV system are decomposed by the EMD to obtain several basic modal components that have little influence on each other. This simplifies the interference or coupling of the characteristic information in the signal sequence and reduces the nonstationarity of the signal. Using this approach, an output power prediction model of a grid-connected PV system is proposed in this paper to optimize the support vector machine (SVM) with the artificial bee colony algorithm.

### 5.1. Optimal Parameter Selection for the SVM Model Based on ABC Algorithm

**Figure 4:**
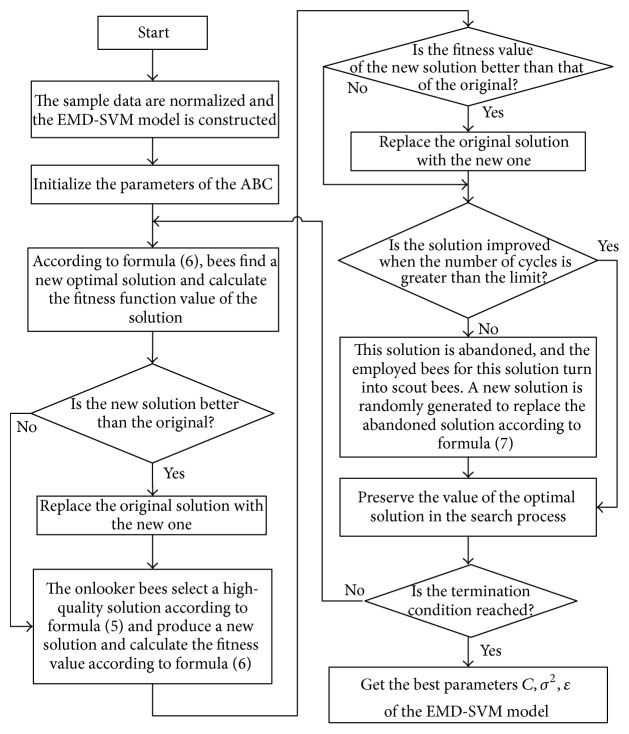
Program flow chart of the SVM parameter optimization using the ABC algorithm.

The penalty coefficient *C*, the insensitivity coefficient *ε*, and the kernel function parameter *σ*^2^ in the SVM determine the accuracy and generalization performance of the algorithm. However, the selection of these three parameters still lacks an effective solution. To address this problem, this paper adopts the artificial bee colony algorithm to optimize the selection of the SVM parameters. The flow diagram of this method is shown in Figure 4.

It is found from several tests that the EMD-SVM prediction model can achieve the ideal prediction accuracy and generalization ability when the parameters of the ABC algorithm are initialized as follows in predicting the PV system output power. The colony size is *N* = 160, the numbers of nectar-gathering and observing bees are both 80, the number of the initial nectar sources (the initial solutions to the optimized problem) is 80, the maximum number of updates of the nectar source is 90, and the maximum number of algorithm loops is 150.

### 5.2. Constructing a Power Prediction Model for a Grid-Connected PV System

**Figure 5:**
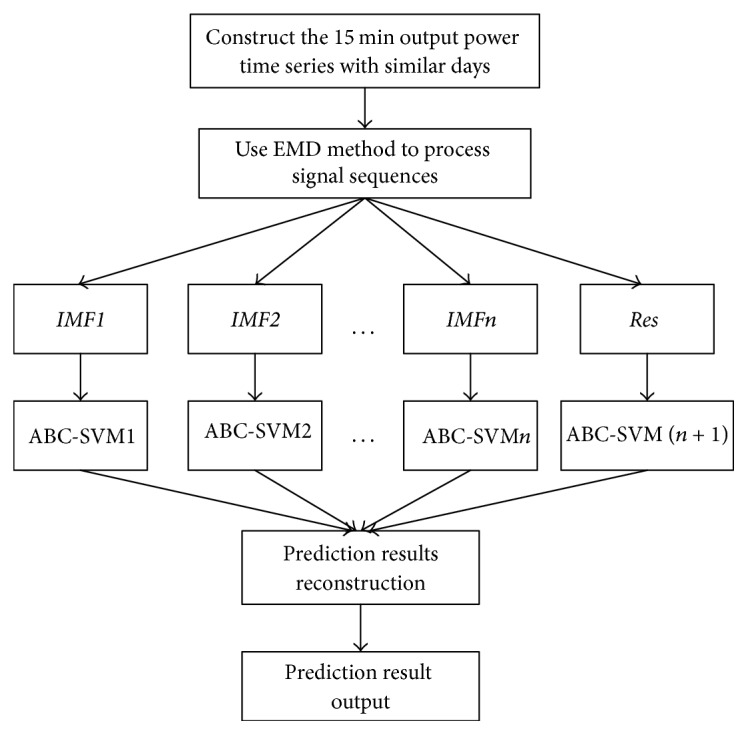
PV system output power forecasting model construction based on the EMD-ABC-SVM.

First, the 15-minute output power time series of similar days is constructed based on the weather forecast data of the prediction day. Then, the output power time series is decomposed using the empirical mode to obtain the intrinsic modal component IMF*n* and the trend component Res at different scales. The corresponding support vector machine prediction models are established for each IMF component and trend component. The input of the model includes the weather type *w*, the maximum temperature *T*^h^, the minimum temperature *T*^l^, the maximum humidity *H*^h^, and the minimum humidity *H*^l^ of the prediction day as well as the corresponding IMF components or corresponding values of the trend component Res for similar days within the last 5 days of the prediction day. The output of the model is the predicted power value. The ABC algorithm optimization of SVM parameters is carried out. Finally, the prediction results of each model are reconstructed to obtain the predicted value of the output power of the grid-connected PV system. The flow chart is shown in Figure 5.

## 6. Simulation Results of the Case Study

### 6.1. Data Preprocessing

To verify the performance of the output power prediction model for a PV system based on EMD and ABC-SVM, the Matlab software is used to complete the model construction. The prediction model is tested and analyzed on a test platform of the 10 kW grid-connected PV system operating in the engineering technology center of a university in Henan province. The sample data sets used in this study are the measured values of the output power of the grid-connected PV system and the local weather data records. In the test, the actual power data in the whole year of 2014 are selected as the research object. The data are classified into five categories according to the weather types, sunny, sunny to cloudy, overcast, and rainy (snowy) and are recorded every 15 minutes. In this example, one day is one period. According to the local sunshine characteristics of Anyang, the summer PV system outputs power for approximately 11 hours per day on average. In this paper, 11 h is chosen for each period, the generated power data are sampled once every 15 min, the meteorological parameters for each period are the temperature and weather type, and the input variable of the model obtained is *X* = (*x*_1_, *x*_2_,…, *x*_*n*_), where *n* is 228, *x*_1_ ~ *x*_220_ represent 220 pieces of data sampled once every 15 minutes on 5 similar days that is closest to the prediction day, *x*_221_ ~ *x*_224_ represent the maximum temperature, minimum temperature, maximum humidity, and minimum humidity of the similar day, and *x*_225_ ~ *x*_228_ represent the maximum temperature, minimum temperature, maximum humidity, and minimum humidity of the prediction day. The output variables of the model are 44 output power values within the prediction day.

According to the method described above, the clustering analysis of similar days is carried out for the weather types in 2014. These days are divided into five typical weather and four seasonal types. In our study, there are 340 sets of sample data (actually, there are 365 sets of data, but 15 of them are bad data) including 208 sets in sunny days, and we will focus on these 208 sets of data firstly. The data of 200 similar days with a sunny weather type is taken as the training data set, and the 8 sets were used as test data: February 24, February 27, May 26, May 28, August 30, August 31, November 25, and November 29. We will establish 4 forecasting models under 4 seasonal types separately and take summer type as an example to introduce the establishment and forecasting process of the model in detail.

**Figure 6:**
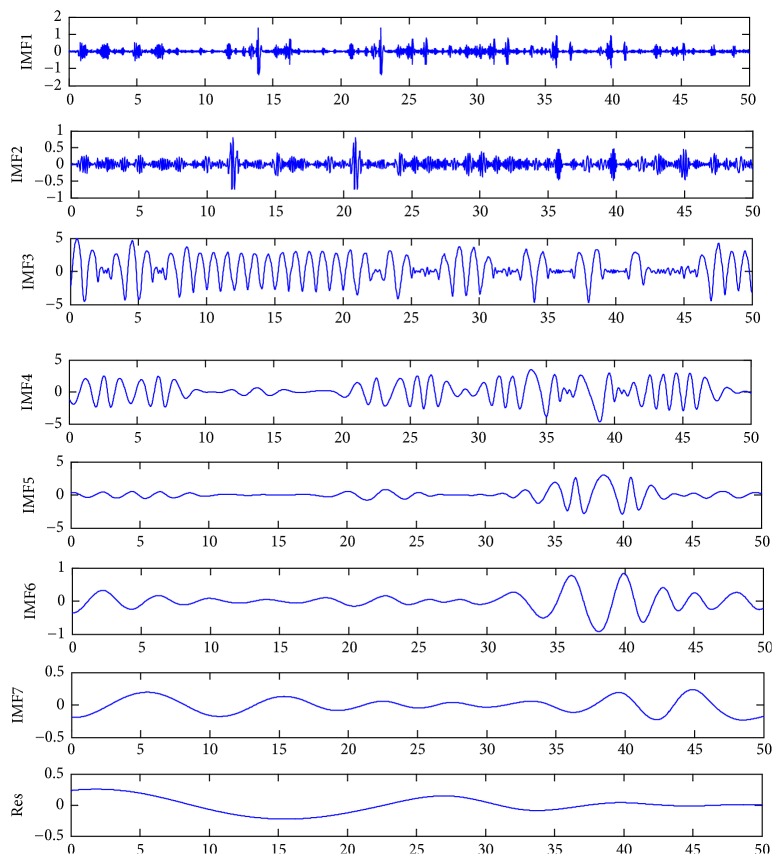
Decomposition graph of the output power sequence of 50 similar days with the EMD.

The model operates according to the construction method for the EMD-ABC-SVM power prediction model in the previous section. First, the EMD decomposition is conducted for the output power sequence of 50 similar days under summer type to obtain seven IMF components and one Res component, as shown in Figure 6. IMF1 and IMF2 are the high-frequency components and have strong nonlinear and random change characteristics caused by abrupt changes in the weather. The frequencies of IMF3–IMF7 become significantly lower and show a strong periodicity, which is affected by meteorological factors; this is the main component of the output power. The residual component Res shows relatively gentle changes, has small amplitude, and is the minor component of the output power.

A SVM power prediction model is constructed for each component, and the parameters of each SVM model are optimized using the artificial bee colony algorithm. The steps are shown in Figure 4. The performance test of the SVM model after parameter optimization can be tested with the test set.

### 6.2. Experiment Results and Discussion

**Figure 7:**
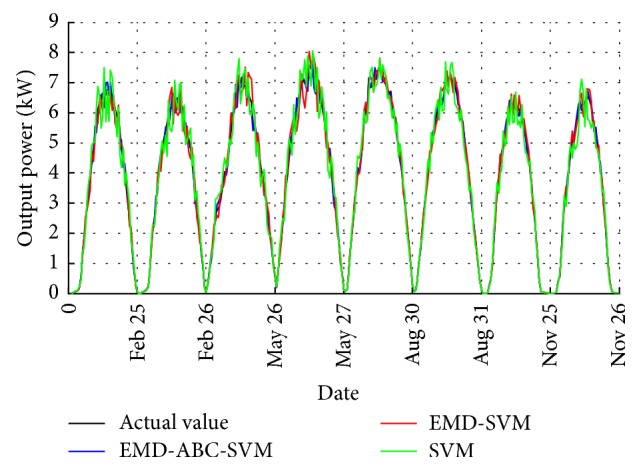
Predicted result curves for the different models.

**Figure 8:**
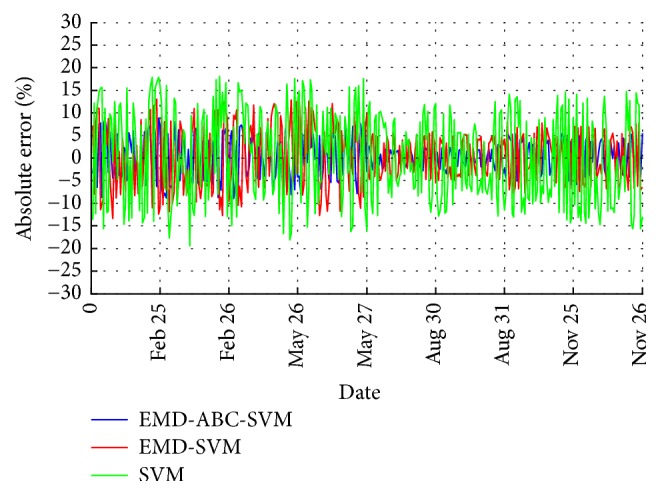
Error curves of different models.

The EMD-ABC-SVM, EMD-SVM, and single SVM models are used to predict the output power of the grid-connected PV system on February 24, February 27, May 26, May 28, August 30, August 31, November 25, and November 29. The prediction results are shown in Figures 7 and 8. Due to limited space, we only give detailed data for two days: August 30th and August 31st, as shown in Tables 1 and 2. In the three models, the EMD-ABC- SVM is several SVM prediction models that have been optimized by the ABC algorithm and constructed by multiple IMF components and an Res component that are obtained through EMD decomposition of the original signal. To verify the effectiveness of the ABC algorithm, we also established an EMD-SVM of the SVM parameters that had not been optimized. The single SVM model predicts the original sequence directly.

By comparing the three types of prediction models, we can see that the EMD-ABC-SVM has the highest accuracy, which indicates that the IMF components after the EMD decomposition reduce the influence of the nonstationarity and randomness in the SVM models and that the parameter optimization of the ABC algorithm gives the best performance for the SVM models. The prediction error in the two periods 7:00 am~9:00 am and 16:00 pm~18:00 pm is relatively large, but the actual amount of electricity generated in the morning and evening is smaller than the total amount of electricity generated throughout the day, indicating that these errors do not affect the practical application of the prediction model.

To fully verify the performance of the model, we have the output power of the grid-connected PV system under the four different weather types: sunny, sunny to cloudy, cloudy, and overcast. The performance of the models is compared and judged with the* MAPE* and* RMSE*. The comparison results of the* MAPE* and* RMSE* for different prediction models are shown in Table 3.

The data given in Table 3 show that the weather types have different effects on the various prediction models. For sunny days, the three models all have good performance, but the EMD-ABC-SVM has the best prediction effect with an MAPE and RMSE of only 6.35% and 7.59%, respectively. In addition, the single SVM model without the EMD decomposition has a prediction error below 15%. For cloudy and sunny to cloudy days, the prediction effects are not ideal. The RMSE of the EMD-ABC-SVM model is up to 14.16%, and the maximum error of the single SVM model without the EMD decomposition is 21.27%. These results are mainly because the cloudy and sunny to cloudy weather conditions change frequently and increase the randomness of the data. The three prediction models have different performances under the same weather type. The prediction error of the EMD-ABC-SVM model is the smallest under the various weather types. The fundamental reason is that the original output power sequence establishes different ABC-SVM models after the EMD decomposition, which reduces the random interference of the power signal and reduces the mutual influence of the characteristic information in the power signal. Additionally, the parameters of the SVM models are optimized with the ABC algorithm to achieve the best working conditions. Therefore, even in cloudy and sunny to cloudy weather conditions with strong randomness, its performance is better than that of the EMD-SVM with nonoptimized parameters and the single SVM that is not decomposed by the EMD.

Grid search and cross validation are usually adopted to optimize parameter of SVM and EMD-SVM. But the grid search is an exhaustive search method and it will take a long time when the range of parameters is large. Optimal parameter selection for EMD-ABC-SVM uses the artificial bee colony algorithm and cross validation. Artificial bee colony algorithm is a heuristic search algorithm, so it did not need all data within the scope of traversal parameters in the group. Furthermore, in the process of optimization, it can use their own individual experience or exchange of experience to change the search strategy, so it can save a lot of time.

In order to apply the PV power prediction method to the practical photovoltaic power generation system, we have developed a PV power forecasting system. The system is developed on Eclipse platform, using Struts2 framework based on MVC model and data persistence framework Hibernate to implement the Web application, using Apache-Tomcat5.5 as Web server. The system mainly includes system management module, data management module, and power prediction module based on EMD-ABC-SVM. The system management module is mainly responsible for the management of the basic user information of the system, such as user information modification, adding or deleting user, and user's rights management. The data management module realizes the management of the power and weather data of the system, such as data import and export, data query, and display. The power prediction module based on EMD-ABC-SVM realizes the short-term prediction of photovoltaic power generation system and saves the prediction results into the database.

At present, the system is running normally, and the practical application proves that the system is practical and the prediction accuracy can meet the actual demand.

The predicting system has played an important role in application. First, the accurate power forecasting can help power dispatching department to make overall arrangements on the optimal combination of conventional power generation and photovoltaic power generation, effectively mitigate the adverse impact of PV power fluctuations on the power grid, and ensure the security and stability of the public grid system. Second, the predicting system can increase utilization efficiency of grid-connected photovoltaic system and help to reduce the spare capacity of the rotating equipment in the thermal power plant and reduce the fuel consumption. Third, the prediction data can provide reference for us to arrange maintenance and overhaul for the photovoltaic array and inverter properly and improve the economic benefits of photovoltaic power station.

## 7. Conclusions

In this paper, the artificial bee colony optimization algorithm and empirical mode decomposition method are combined and successfully applied to the field of short-term prediction of the output power of a grid-connected PV system. Similar days of the same season are filtered with the fuzzy *C*-mean. The EMD method is used to conduct the empirical modal decomposition of the output power series, producing the intrinsic modal component IMF under *n* different scales and one trend component Res. The corresponding SVM prediction model is established for each component, and the optimizing pretreatment using the artificial bee colony algorithm is done for the SVM model parameters. Finally, the results of each prediction model are integrated and reconstructed to obtain the predicted values of output power from the PV system. The results acquired from the test using measured data show that the effect of the EMD-ABC-SVM prediction model is superior to those of the single SVM prediction model and the unoptimized EMD-SVM prediction model. The proposed method improves the prediction accuracy of output power of the grid-connected PV system, reduces the influence of randomness of the PV generated power on the safe and reliable operation of the public power grid, and provides an effective method for the optimal scheduling of the output grid power.

## Supplementary Material

KKT-in-SVM.

## Figures and Tables

**Table 1:** Comparisons of predicted results using three models about output power data of August 30 and 31.

Number	Observed value (kW)	EMD-ABC-SVM (kW)	EMD-SVM (kW)	SVM (kW)
1	0.0683	0.0696	0.0657	0.0757
2	0.1382	0.1420	0.1317	0.1555
3	0.6275	0.6243	0.6521	0.5683
4	1.3239	1.2890	1.3952	1.1614
5	1.8403	1.8726	1.7653	1.9942
6	2.4590	2.5307	2.5803	2.6949
7	3.0621	3.1141	3.2043	2.6729
8	3.5830	3.5301	3.4405	3.9371
9	4.1412	4.1757	3.9426	4.5710
10	4.1223	4.0149	4.0081	4.4794
11	4.5165	4.4380	4.6674	4.8161
12	4.9385	5.0365	4.7940	5.3263
13	5.7823	5.8514	6.0638	6.0453
14	6.1282	6.0760	6.3411	5.6505
15	5.9762	6.0834	5.7404	5.7478
16	7.0898	7.1569	6.7795	6.4695
17	6.8198	6.8784	7.2080	7.3588
18	6.8953	7.0247	7.0808	7.5045
19	7.0273	7.1157	6.6779	6.6845
20	7.3684	7.4928	7.1133	6.9091
21	7.1951	7.1152	7.4001	6.8981
22	7.4464	7.3458	7.2335	7.2037
23	7.5219	7.6221	7.8194	7.7988
24	7.3848	7.2940	7.1768	7.1231
25	7.1722	7.0326	7.4188	7.5909
26	7.1411	7.0819	6.9770	7.3482
27	6.9242	6.9742	7.2109	7.2068
28	6.6868	6.7990	6.8926	6.4834
29	6.6450	6.6780	6.4818	6.5732
30	6.7188	6.5734	6.5100	6.2542
31	6.2262	6.1659	6.3334	5.8241
32	5.8366	5.9725	6.1233	6.1434
33	5.8367	5.7635	5.6231	5.3474
34	5.2529	5.1492	5.3918	5.6716
35	4.8899	4.9357	4.6674	4.5165
36	4.5304	4.4036	4.7096	4.9729
37	4.0192	4.1235	4.2186	4.4935
38	3.4722	3.5371	3.3074	3.6159
39	3.0201	3.0917	3.1672	3.3319
40	2.5275	2.4921	2.6674	2.2997
41	1.9505	1.8951	1.8598	1.7173
42	1.3713	1.3998	1.4510	1.2572
43	0.8438	0.8691	0.8766	0.9380
44	0.2911	0.2892	0.2753	0.3263
45	0.0710	0.0719	0.0689	0.0649
46	0.1209	0.1186	0.1150	0.1055
47	0.4057	0.3958	0.4239	0.3581
48	0.9364	0.9579	0.9669	0.8624
49	1.4869	1.4462	1.5598	1.6322
50	2.0503	1.9971	2.0131	1.8001
51	2.5964	2.5070	2.6832	2.2883
52	3.0432	3.0052	2.9090	2.8849
53	3.5690	3.6520	3.5014	3.9870
54	4.1105	4.1658	3.9154	4.4732
55	4.5235	4.4440	4.7406	4.0573
56	4.9307	4.8474	4.6929	5.3496
57	5.3317	5.3907	5.1662	4.9708
58	5.5464	5.4451	5.3218	5.8648
59	5.8247	5.9263	5.6195	6.1471
60	6.0815	6.1966	5.8291	6.4295
61	6.2005	6.1257	6.4180	5.7553
62	6.4994	6.4375	6.8183	6.8420
63	6.3893	6.2739	6.7277	6.0532
64	6.5374	6.6386	6.8621	6.0958
65	7.2982	7.1788	7.0188	7.6870
66	7.1500	7.2463	7.3755	6.7295
67	7.1444	7.0291	6.8737	6.6131
68	7.1668	7.2924	7.3184	7.5403
69	7.1329	7.0655	6.9848	7.6720
70	6.9410	6.8737	6.6731	7.3374
71	6.8850	7.0130	7.1719	6.3297
72	6.6407	6.7612	6.9030	6.1948
73	6.3970	6.2742	6.6972	6.1108
74	6.1954	6.2463	5.9181	5.7215
75	5.8939	5.8214	6.1064	6.1097
76	5.6886	5.5888	5.5214	5.2973
77	5.2085	5.2400	4.9992	4.7936
78	4.7334	4.8256	4.8872	4.4592
79	4.3456	4.3096	4.5047	4.1997
80	3.7633	3.8380	3.9432	3.4130
81	3.3305	3.2800	3.4314	3.5489
82	2.8878	2.8068	3.0318	3.2491
83	2.3862	2.4197	2.2780	2.1935
84	1.8173	1.8728	1.8669	1.5836
85	1.3833	1.4221	1.3180	1.5195
86	0.9191	0.9363	0.9614	1.0168
87	0.4481	0.4326	0.4613	0.4160
88	0.1267	0.1237	0.1320	0.1121

**Table 2:** Comparisons of the three models about forecast accuracy using output power data of August 30 and 31.

Number	EMD-ABC-SVM (%)	EMD-SVM (%)	SVM (%)
1	1.893	−3.873	10.729
2	2.725	−4.746	12.530
3	−0.518	3.921	−9.442
4	−2.636	5.385	−12.277
5	1.755	−4.077	8.363
6	2.919	4.933	9.595
7	1.697	4.642	−12.710
8	−1.478	−3.977	9.881
9	0.834	−4.796	10.379
10	−2.607	−2.772	8.662
11	−1.739	3.340	6.633
12	1.985	−2.925	7.853
13	1.194	4.867	4.548
14	−0.851	3.474	−7.795
15	1.795	−3.946	−3.821
16	0.946	−4.376	−8.750
17	0.859	5.691	7.903
18	1.877	2.691	8.834
19	1.258	−4.973	−4.879
20	1.689	−3.462	−6.233
21	−1.110	2.850	−4.127
22	−1.350	−2.859	−3.259
23	1.332	3.954	3.681
24	−1.230	−2.817	−3.544
25	−1.946	3.439	5.839
26	−0.829	−2.299	2.900
27	0.723	4.141	4.082
28	1.678	3.077	−3.042
29	0.497	−2.455	−1.079
30	1.893	−3.873	10.729
31	−2.165	−3.108	−6.915
32	−0.968	1.722	−6.458
33	2.328	4.912	5.256
34	−1.254	−3.659	−8.383
35	−1.974	2.644	7.970
36	0.937	−4.550	−7.637
37	−2.799	3.956	9.768
38	2.594	4.962	11.800
39	1.870	−4.746	4.138
40	2.369	4.870	10.324
41	−1.401	5.536	−9.013
42	−2.842	−4.651	−11.955
43	2.074	5.810	−8.320
44	2.991	3.880	11.157
45	−0.660	−5.452	12.081
46	1.322	−3.001	−8.557
47	−1.917	−4.930	−12.766
48	−2.434	4.487	−11.730
49	2.301	3.256	−7.906
50	−2.741	4.900	9.771
51	−2.597	−1.815	−12.205
52	−3.446	3.341	−11.868
53	−1.249	−4.409	−5.201
54	2.326	−1.894	11.712
55	1.346	−4.745	8.824
56	−1.758	4.800	−10.306
57	−1.689	−4.823	8.495
58	1.108	−3.103	−6.769
59	−1.828	−4.050	5.740
60	1.744	−3.524	5.535
61	1.893	−4.150	5.723
62	−1.206	3.507	−7.180
63	−0.953	4.906	5.270
64	−1.805	5.296	−5.260
65	1.549	4.967	−6.754
66	−1.636	−3.828	5.328
67	1.346	3.154	−5.882
68	−1.614	−3.790	−7.437
69	1.753	2.116	5.213
70	−0.945	−2.076	7.558
71	−0.969	−3.859	5.712
72	1.859	4.166	−8.065
73	1.816	3.950	−6.715
74	−1.920	4.693	−4.474
75	0.821	−4.477	−7.649
76	−1.229	3.606	3.662
77	−1.754	−2.940	−6.879
78	0.605	−4.019	−7.967
79	1.948	3.249	−5.795
80	−0.827	3.661	−3.358
81	1.987	4.782	−9.306
82	−1.515	3.029	6.557
83	−2.804	4.987	12.512
84	1.406	−4.533	−8.073
85	3.059	2.732	−12.86
86	2.807	−4.719	9.852
87	1.865	4.598	10.621
88	−3.470	2.945	−7.167

**Table 3:** Prediction error and running time comparison between different models and different weather types.

Weather types	EMD-ABC-SVM		EMD-SVM		SVM	
	MAPE/%	RMSE/%	MAPE/%	RMSE/%	MAPE/%	RMSE/%
Sunny	6.35	7.59	9.73	10.46	12.89	13.74
Sunny to cloudy	12.38	13.05	15.26	17.03	18.44	20.63
Cloudy	13.55	14.16	17.03	18.52	19.68	21.27
Overcast	10.89	12.07	13.95	14.83	16.66	18.72

## References

[1] Sabo M. L., Mariun N., Hizam H., Mohd Radzi M. A., Zakaria A. (2016). Spatial energy predictions from large-scale photovoltaic power plants located in optimal sites and connected to a smart grid in Peninsular Malaysia. *Renewable and Sustainable Energy Reviews*.

[2] Yao Z., Yu F., Zhao Q. (2013). Simulation research on large-scale PV grid-connected systems based on MMC. *Proceedings of the CSEE*.

[3] Chen Q., Li L.-D., Wang Q.-J. (2013). Simulation model of photovoltaic generation grid-connected system and its impacts on voltage stability in distribution grid. *Transactions of China Electrotechnical Society*.

[4] Song X.-H., Guo Z.-Z., Guo H.-P. (2015). A new forecasting model based on forest for photovoltaic power generation. *Power System Protection and Control*.

[5] Gulin M., Pavlović T., Vašak M. (2017). A one-day-ahead photovoltaic array power production prediction with combined static and dynamic on-line correction. *Solar Energy*.

[6] Zhi Y.-G. (2015). Output power prediction of photovoltaic system based on time-series measurement Data. *Journal of Quality Engineering Society*.

[7] Gulin M., Pavlović T., Vašak M. (2016). Photovoltaic panel and array static models for power production prediction: integration of manufacturers' and on-line data. *Renewable Energy*.

[8] Rana M., Koprinska I., Agelidis V. G. (2016). Univariate and multivariate methods for very short-term solar photovoltaic power forecasting. *Energy Conversion and Management*.

[9] Bai J.-L., Mei H.-W. (2014). Improved similarity based fuzzy clustering algorithm and its application in the PV array power short term forecasting. *Power System Protection and Control*.

[10] Khademi M., Moadel M., Khosravi A. (2016). Power prediction and technoeconomic analysis of a solar PV power plant by MLP-ABC and COMFAR III, considering cloudy weather conditions. *International Journal of Chemical Engineering*.

[11] Dang X. J., Chen H. Y., Jin X. M. (2013). A method for forecasting short-term wind speed based on EMD and SVM. *Applied Mechanics and Materials*.

[12] Selakov A., Cvijetinović D., Milović L., Mellon S., Bekut D. (2014). Hybrid PSOSVM method for short-term load forecasting during periods with significant temperature variations in city of Burbank. *Applied Soft Computing*.

[13] Chen R., Liang C.-Y., Hong W.-C., Gu D.-X. (2015). Forecasting holiday daily tourist flow based on seasonal support vector regression with adaptive genetic algorithm. *Applied Soft Computing Journal*.

[14] Gao X.-M., Liu F.-B., Yang S.-F. (2013). Intelligent fault diagnosis of water supply network based on ELM. *Computer Engineering and Design*.

[15] Bipin P., Rao P. (2016). Linearization of high power amplifier using modified artificial bee colony and particle swarm optimization algorithm. *Procedia Technology*.

[16] Shi Y., Pun C.-M., Hu H., Gao H. (2016). An improved artificial bee colony and its application. *Knowledge-Based Systems*.

[17] Mohammadi R., Javidan R. (2016). An intelligent traffic engineering method over software defined networks for video surveillance systems based on artificial bee colony. *International Journal of Intelligent Information Technologies*.

[18] Krishnamoorthy M., Umapathy P. (2016). Hybrid voltage control model for hybrid renewable energy system using artificial bees colony algorithm. *Circuits and Systems*.

[19] Xu D., Yu L., Wang Y. (2014). Mechanical fault diagnosis method for circuit breaker based on the combination of energy entropy and improved EMD by using the least square method. *High Voltage Apparatus*.

[20] Zang H.-G., Li Q.-Z. (2014). Application of improved EMD method on extraction of partial discharge signal. *Proceedings of the CSU-EPSA*.

[21] Lu W., Zhang L., Liang W., Yu X. (2016). Research on a small-noise reduction method based on EMD and its application in pipeline leakage detection. *Journal of Loss Prevention in the Process Industries*.

[22] Wang Y., Wu X., Li W., Li Z., Zhang Y., Zhou J. (2016). Analysis of micro-doppler signatures of vibration targets using EMD and SPWVD. *Neurocomputing*.

[23] Wang S., Zhang N., Wu L., Wang Y. (2016). Wind speed forecasting based on the hybrid ensemble empirical mode decomposition and GA-BP neural network method. *Renewable Energy*.

[24] Karaboga D., Ozturk C. (2011). A novel clustering approach: artificial bee colony (ABC) algorithm. *Applied Soft Computing Journal*.

