# Supplementary material for: Optimal Parameter Selection for Support Vector Machine Based on Artificial Bee Colony Algorithm: A Case Study of Grid-Connected PV System Power Prediction
Source: Comput Intell Neurosci. 2017 Aug 22;2017:7273017. doi: 10.1155/2017/7273017 (PMC5585556; doi:10.1155/2017/7273017)
Supplement: Supplementary file 1 — KKT-in-SVM. [file 7273017.f1.doc]

**supporting information**

**How is KKT condition solved in SVM？**

Support vector machine can minimize the expected error and overcome the problem of overfitting because it is based on structural risk minimization principle. According to previous research, SVM has been proved to provide better resolutions for both classification and regression in different fields: fault classification, electricity load forecasting, wind speed forecasting, prediction of the air quality and so on. The basic principles of SVM to solve regression prediction problems are described as follows.

The sample set is normally denoted as：

$X=\left\{(x_{i},y_{i})|x\in R^{n},\text{ }y\in R,\text{ }i=1,2,\cdots,n\right\}$ （1）

The regression model defines the functional relationship between *x_i_* and *f*(*x_i_*) as:

$y=f(x_{i})=w\cdot x_{i}+b$ (2)

where *w*, *b* are the weight vector and threshold respectively.

According to the risk minimization principle, solving regression functio becomes the objective function minimization problem:

$\min\left\{\frac{1}{2}\left\|w^{2}\right\|+C\sum_{i=1}^{l} (\xi_{i}^{*}+\xi_{i})\right\}$ (3)

$$St\text{ }\left\{\begin{matrix} y_{i}-w\bullet x_{i}-b\leq\varepsilon+\xi_{i}^{*} \\ w\bullet x_{i}+b-y_{i}\leq\varepsilon+\xi_{i} \\ \text{ }\xi_{i}^{*}\text{, }\xi_{i}\geq0;i=1,\text{ }2...\text{ }l \end{matrix}\right.$$

where *C* is penalty coefficient, $\xi_{i}^{(*)}$ is slack variable, *ε* is insensitive loss function. $\xi_{i}^{(*)}$ guar- antees the satisfaction of constraint condition; *C* controls the equilibrium between the complexity of model and training error; *ε* is a preset constant that for controlling tube size. If *ε* is set too small, it will lead to overfitting, otherwise, it is easy to lead to the underfitting..

For nonlinear regression, assume that there is such a transform: $\Phi:\text{ }R^{n}\to H,\text{ }x\to\Phi(x)$, so Eq.3 can be reformulated as:

$\min\left\{\frac{1}{2}\left\|w^{2}\right\|+C\sum_{i=1}^{l} (\xi_{i}^{*}+\xi_{i})\right\}$ （4）

$$St\text{ }\left\{\begin{matrix} y_{i}-w\bullet\Phi(x_{i})-b\leq\varepsilon+\xi_{i}^{*} \\ w\bullet\Phi(x_{i})+b-y_{i}\leq\varepsilon+\xi_{i} \\ \text{ }\xi_{i}^{*}\text{, }\xi_{i}\geq0;i=1,\text{ }2...\text{ }l \end{matrix}\right.\alpha^{(*)}$$

where *x_i_* is mapped to a higher dimensional feature space by function $\Phi$. By performing the mapping, we hopes that the nonlinear regression problem in the x-space will be converted into a linear regression problem in the $\Phi$space.

Since $\Phi$ may map *x_i_* to a very high or infinite dimensional space and numerical optimization in a high-dimensional space suffers from the curse of dimensionality. So we can solve Lagrange multiplier vector $\alpha^{(*)}=(\alpha_{1},\alpha_{1}^{*},\cdots,\alpha_{l},\alpha_{l}^{*})^{T}$ for the dual problem of Eq. 4 instead of solving *w* for Eq. 4.

In order to obtain the dual problem of Eq. 4, we introduce the Lagrange function as follows:

$\begin{matrix} L=\frac{1}{2}\left\|w\right\|^{2}+C\sum_{i=1}^{l} (\xi_{i}^{*}+\xi_{i})-\sum_{i=1}^{l} (\eta_{i}^{*}\xi_{i}^{*}+\eta_{i}\xi_{i})-\sum_{i=1}^{l} \alpha_{i}(\varepsilon+\xi_{i}+y_{i}-w\bullet\Phi(x_{i})-b) \\ \text{ }-\sum_{i=1}^{l} \alpha_{i}^{*}(\varepsilon+\xi_{i}^{*}-y_{i}+w\bullet\Phi(x_{i})+b) \end{matrix}$ (5)

First, the partial derivative *L* sub *w*, *b*, *ξ_i_*, *ξ_i_^*^* is calculated separately:

(6)

Then the dual problem of Eq.4 can be obtained as following:

$St\text{ }\left\{\begin{matrix} \sum_{i=1}^{l} (a_{i}^{*}-a_{i})=0 \\ a_{i}^{*},a_{i}\in[0,C],i=1,2...,l \end{matrix}\right.$ (7)

This is an optimization problem in Euclidean space $R^{2l}$, so it has solutions:

$\alpha^{(*)}=(\alpha_{1},\alpha_{1}^{*},\cdots,\alpha_{l},\alpha_{l}^{*})^{T}$ (8)

From Eq. 6 we can know:

$w=\sum_{i=1}^{l} (\alpha_{i}^{*}-\alpha_{i})\Phi(x_{i})$ (9)

Using KKT（Karush-Kuhn -Tucker）conditions:

$\alpha_{j}(\varepsilon_{j}+\xi_{j}-y_{j}+(w\bullet\Phi(x_{j}))+b)=0$ （10）

$\alpha_{j}^{*}(\varepsilon_{j}^{*}+\xi_{j}^{*}+y_{j}-(w\bullet\Phi(x_{j}))-b)=0$ （11）

$(C-\alpha_{j})\xi_{j}=0$ (12)

$(C-\alpha_{j}^{*})\xi_{j}^{^{*}}=0$ (13)

When $\alpha_{j}$≠0，There must be $\varepsilon_{j}+\xi_{j}-y_{j}+(w\bullet\Phi(x_{j}))+b=0$; when $\alpha_{j}^{*}$≠0，There must be $\varepsilon_{j}^{*}+\xi_{j}^{*}+y_{j}-(w\bullet\Phi(x_{j}))-b=0$; for any data point $\varepsilon_{j}^{(*)}>0$, so It is impossible that $\alpha_{j}$ and $\alpha_{j}^{*}$are not zero at the same time.

Therefore, according to Eq. 12 and Eq. 13, there must be $\xi_{j}$=0 , $\xi^{*}_{j}$=0 and:

$b=y_{j}-\varepsilon_{j}-(w\bullet\Phi(x_{j}))=y_{j}-\varepsilon_{j}-\sum_{i=1}^{l} (\alpha_{i}^{*}-\alpha_{i})\Phi(x_{i})\bullet\Phi(x_{j})\text{ }$ (14)

The nonlinear regression function can be determined:

$y=f(x)=\sum_{i=1}^{l} (a_{i}^{*}-a_{i})\Phi(x_{i})\bullet\Phi(x)+b$ (15)

$$S.t.\text{ }\left\{\begin{matrix} \sum_{i=1}^{l} (a_{i}^{*}-a_{i})=0 \\ 0\leq a_{i}^{(*)}\leq C,\text{ }i=1,\text{ }\cdots,\text{ }l \end{matrix}\right.$$

making $K(x,x')=\Phi(x)\cdot\Phi(x')$, where $K(x,x')$ is called kernel function and () denotes inner product operation. When a $K(x,x')$ satisfies the Mercer condition, it corresponds to the inner product of a transform space according to the functional theory. In this study, We chose Gaussian radial basis func- tion as the kernel function：

$K(x,x')=\exp\left(-\left.\left|x-x'\right.\right|^{2}\right)/2\sigma^{2}$ (16)

So the nonlinear regression function can be expressed as:

$y=f(x)=\sum_{i=1}^{l} (a_{i}^{*}-a_{i})K(x_{i},x)+b$ (17)

$$S.t.\text{ }\left\{\begin{matrix} \sum_{i=1}^{l} (a_{i}^{*}-a_{i})=0 \\ 0\leq a_{i}^{(*)}\leq C,\text{ }i=1,\text{ }\cdots,\text{ }l \end{matrix}\right.$$

where $\sigma$ is the kernel parameter, and it precisely defines the structure of high dimensional feature space. The penalty coefficient *C*, the insensitivity coefficient $\varepsilon$ and the kernel function parameter $\sigma$in SVM determine the accuracy and genera- lization performance of the algorithm.
